# TiO_2_-embedded molecularly imprinted polymer as electrochemical sensor for ultrasensitive determination of glycopyrronium bromide

**DOI:** 10.5599/admet.3102

**Published:** 2025-12-08

**Authors:** Zehra Dogan, Ensar Piskin, Ahmet Cetinkaya, Esen Bellur Atici, Sibel A. Ozkan

**Affiliations:** 1 Ankara University, Faculty of Pharmacy, Department of Analytical Chemistry, Ankara, Türkiye; 2 Ankara University, Graduate School of Health Sciences, Ankara, Türkiye; 3 University of Health Sciences, Gülhane Faculty of Pharmacy, Department of Analytical Chemistry, Ankara, Türkiye; 4 Hacettepe University Faculty of Pharmacy, Department of Analytical Chemistry, Ankara, Türkiye; 5 Gebze Technical University, Department of Chemistry, Kocaeli, Türkiye; 6 DEVA Holding A.S., R&D Center, Tekirdağ, Türkiye

**Keywords:** Electrochemical detection, photopolymerization method, glassy carbon electrode, drug analysis, nanomaterial, real sample

## Abstract

**Background and purpose:**

The precise quantification and quality evaluation of glycopyrronium bromide (GLB), a long-acting muscarinic antagonist widely used in the treatment of chronic obstructive pulmonary disease, requires the development of advanced analytical methodologies capable of achieving high sensitivity, accuracy, and selectivity to ensure therapeutic efficacy and formulation integrity. This study aims to overcome the limitations of conventional methods by developing a rapid, cost-effective method for determining GLB.

**Experimental approach:**

To achieve this, titanium dioxide nanoparticles (TiO_2_ NPs) were initially applied onto a glassy carbon electrode surface to provide an enhanced surface area and increased conductivity. Subsequently, a TiO_2_ nanoparticle-supported molecularly imprinted polymer (MIP) film was synthesized via photopolymerization using GLB as the template molecule, 4-amminobenzoic acid (4-ABA) as the functional monomer, ethylene glycol dimethacrylate as the crosslinking agent, and 2-hydroxyethyl methacrylate (HEMA) as the basic monomer.

**Key results:**

The optimized GLB/4-ABA@TiO_2_ NPs/MIP- sensor demonstrated outstanding analytical performance, achieving ultra-low picomolar detection limits. The system exhibited superior selectivity (confirmed by high imprinting factor), excellent repeatability and reproducibility, and satisfactory stability. It was successfully applied to the accurate measurement of GLB in both commercial serum and pharmaceutical formulations.

**Conclusion:**

The designed nanomaterial-embedded MIP-based electrochemical system presented here offers a highly successful, sensitive, and selective method for GLB determination. The work significantly advances knowledge in the field of analytical medicine and drug monitoring by providing a fast, robust alternative for routine clinical and quality-control tracking of GLB.

## Introduction

Chronic obstructive pulmonary disease (COPD) is defined by persistent respiratory symptoms and irreversible, slowly progressive airflow limitation, representing a major global health problem associated with high morbidity and mortality [1]. The Global Initiative for Chronic Obstructive Lung Disease (GOLD) guidelines recommend that patients with stable COPD use long-acting bronchodilators, specifically long-acting muscarinic antagonists (LAMAs), to improve lung function and alleviate symptoms [2]. Glycopyrronium bromide (GLB) is a key LAMA with a quaternary ammonium structure. This anticholinergic agent counteracts the bronchoconstrictor effect of acetylcholine by competitively inhibiting muscarinic receptors, thereby inducing bronchodilation. GLB is newly licensed as a maintenance bronchodilator treatment for COPD. While current GLB products are often delivered via dry-powder inhalation systems, formulating alternative devices, such as pressurized metered-dose inhalers (pMDIs), is challenging due to the complex formulation issues associated with them. Given the critical role of GLB in therapy and the complexity of its formulative delivery, highly accurate and efficient analytical methods for its quantitative determination in various samples, including pharmaceutical preparations and biological fluids, are essential [3,4].

Numerous analytical techniques have been employed to determine GLB in biological fluids and pharmaceutical formulations. These include diverse separation and detection methods such as high-performance liquid chromatography (HPLC) [5], HPLC-mass spectrometry (HPLC-MS) [6], gas chromategraphy (GC) [7], reversed-phase HPLC with UV detection (RP-HPLC/UV) [8], and spectrophotometry [9]. While each of these methods offers certain advantages, they may have limitations in detecting low-level analytes in complex biological matrices. These techniques require knowledgeable operators to manage a variety of samples and to perform time-consuming, arduous pretreatment steps. Furthermore, they require substantial time and are unsuitable for *in situ* detection.

In contrast to the approaches above, electrochemical techniques have attracted significant attention due to their excellent selectivity, high sensitivity, short analysis times, and simplicity. Because conventional electrochemical sensors can respond to multiple analytes, they often encounter selectivity issues that can lead to false positives or inaccurate readings. This presents significant challenges in drug analysis, especially when working with complex matrices such as biological fluids and mixed drug formulations. Molecularly imprinted polymers (MIPs) are used to address this problem by incorporating a component that detects and discriminates the target analyte into the electrochemical sensor design. MIPs are artificial antibody mimics that significantly increase sensor sensitivity and selectivity while overcoming the stability and cost constraints associated with biological materials [10-12]. However, conventional MIP structures have several significant limitations. These include the heterogeneous distribution of binding sites, the limited number of specific recognition sites available, limited access of the template molecule to these sites, slow and inefficient template removal or rebinding processes, and inadequate electrical conductivity. To overcome these drawbacks, the functional performance of MIPs has been enhanced in recent years by combining them with nanomaterials that possess superior physicochemical properties, such as conductivity, mechanical strength, and a large specific surface area. These hybrid structures not only yield a more homogeneous distribution of binding sites but also enhance rebinding kinetics by enabling faster, more efficient access to analytes. Furthermore, the high surface area and enhanced conductivity provided by nanomaterials significantly improve the overall sensitivity, stability, and reproducibility of sensor platforms, enabling the more reliable and effective use of MIP-based sensors in complex matrices [13-17].

This study presents an electrochemical MIP sensor using titanium dioxide nanoparticles (TiO_2_ NPs) as a supporting material for the sensitive and selective determination of GLB. Drop casting and photopolymerization (PP) methods were used to create the MIP structure on the glassy carbon electrode (GCE) surface, and 4-aminobenzoic acid (4-ABA) was used as the functional monomer, 2-hydroxymethyl methacrylate (HEMA) as the basic monomer, and ethylene glycol dimethacrylate (EGDMA) as the crosslinking agent. The strong non-covalent contacts between the functional groups of GLB and the template molecule produce stable, consistent, and flexible MIP structures. An extensive optimization process was followed before the sensor was used to determine GLB in commercial serum samples and standard solutions. The developed nanomaterial-assisted MIP-based electrochemical sensor demonstrates significant potential as a reliable analytical tool for clinical and pharmaceutical monitoring of GLB.

## Experimental

### Reagents and chemicals

All solutions were prepared using analytical grade chemicals and ultrapure water (MilliQ). GLB (≥99.5 %) used in electrochemical studies was supplied by DEVA Holding A.Ş. (Istanbul, Türkiye). Potassium ferrocyanide (K_3_[Fe(CN)_6_], ≥98.5 %), ferricyanide (K_4_[Fe(CN)_6_], ≥99.0 %), acetic acid (HAc), acetone, acetonitrile (ACN), hydrochloric acid (HCl), methanol (MeOH), ethanol (EtOH), HEMA (≥ 99.9 %), EGDMA (>98.0 %), polyvinyl alcohol (PVA, ≥85.0 %), 2-hydroxy-2-methyl propiophenone (≥97 %), and sodium hydroxide (NaOH, >98.0 %) were purchased from Merck (Darmstadt, Germany). A 2 mg mL^-1^ stock solution of TiO_2_ NPs was prepared in distilled water and dispersed using a sonication device. 4-ABA (1.0 mM, 4-aminobenzoic acid) was prepared. MeOH was used to prepare the GLB (1.0 mM) stock solution. All stock and working solutions used in the experiments were freshly prepared, stored at approximately 4 °C, and renewed weekly.

### Apparatus

Electrochemical measurements, such as cyclic voltammetry (CV) and differential pulse voltammetry (DPV), were performed using an Ivium potentiostat (Eindhoven, the Netherlands). AUTOLAB (Nova 2.1.5 software, The Netherlands) was used for electrochemical impedance spectroscopy (EIS) analysis. In the three-electrode electrochemical configuration, an Ag/AgCl electrode (in 3 M KCl) was used as the reference electrode, a GCE as the working electrode, and a platinum wire as the counter electrode. Weighing was performed with a precision balance (Ohaus Instruments, Shanghai, China), and pH adjustment was performed with a pH meter (Mettler-Toledo pH/ionS220, Greifensee, Switzerland). PP was carried out using a 100 W UV lamp emitting at 365 nm, whereas the rebinding and template-removal processes of the fabricated GLB/4-ABA@TiO_2_ NPs/MIP-GCE were performed using a Thermo-shaker (Biosan TS-100, Riga, Latvia). All experimental procedures were conducted under ambient laboratory conditions. The morphological characterization of the fabricated sensor was performed using scanning electron microscopy (SEM) (GeminiSEM 500, Zeiss, Oberkochen, Germany), and a complementary surface composition analysis was conducted using energy-dispersive X-ray spectroscopy (EDX, Bruker, Berlin, Germany).

### Fabrication of the GLB/4-ABA@TiO_2_ NPs/MIP-GCE sensor

Before the PP process, the GCE surface was mechanically polished using an alumina slurry on a polishing pad, thoroughly rinsed with ultrapure water, and subsequently dried at room temperature. Thereafter, 10 μL of GLB (template molecule, 1.0 mM) and 30 μL of 4-ABA (functional monomer, 1.0 mM) were transferred into an Eppendorf tube and vortexed for 1 min to ensure homogeneous mixing and pre-complex formation between the template and monomer molecules. Then, 50 μL of HEMA (basic monomer) and 10 μL of EGDMA (crosslinker) were added to the mixture, which was then ultrasonicated for 10 min to form a homogeneous solution. 20 μL of the resulting monomer solution was transferred to a separate Eppendorf tube, and 2 μL of the photoinitiator (2-hydroxy-2-methylpropiophenone) was added to the mixture. The GCE surface was coated with 0.25 μL of polymerization solution and then exposed to ultraviolet irradiation (365 nm, 100 W) for 5 min to initiate polymerization. The resulting polymeric film was then allowed to stabilize at room temperature for at least 15 min. Template removal from the developed MIP-based polymeric structure was carried out using a Thermo-shaker (650 rpm, 25 °C) with 15 M HAc as the desorption agent. For the rebinding study, a defined concentration of GLB (10^-11^ M) was incubated on the Thermo-shaker for 10 min. Control experiments were performed under identical conditions using non-imprinted polymers (NIPs) synthesized according to the same procedure, but in the absence of the template molecule.

### Analysis of GLB in capsule dosage form and commercial serum samples

The practical applicability of the developed sensor in capsule dosage systems was assessed by analysing five capsules, each labelled to contain 4 mg of GLB. The gross weights of the filled capsules were first determined, followed by the weighing of empty shells to calculate the net drug content per capsule. The powder obtained from the capsules was homogenized by crushing them in a glass mortar and pestle. The capsule stock solution (1.0 mM) was prepared in MeOH and sonicated for 30 min. The capsule samples were then centrifuged to remove insoluble excipients, yielding a clear supernatant, which was diluted with MeOH to prepare recovery solutions.

Commercial serum samples were stored at -20 °C to prevent enzymatic degradation and chemical deterioration prior to analysis. A stock serum solution was prepared according to established protocols to ensure consistent matrix conditions for recovery experiments. For the determination of GLB recovery, 3.6 mL of commercial serum was transferred into a test tube and diluted with 5.4 mL of acetonitrile to precipitate serum proteins, followed by the addition of 1.0 mL of GLB standard solution (0.1 mM). The mixture was sonicated for 15 min to promote complete homogenization and facilitate efficient interaction between the analyte and the solvent. Subsequently, the samples were centrifuged at 5000 rpm for 25 min to remove precipitated protein residues, yielding a clear supernatant suitable for quantitative analysis. The resulting solutions were then used for recovery studies, enabling the evaluation of the sensor’s accuracy and precision in a complex biological matrix. The obtained supernatant was subsequently diluted to a series of intermediate concentrations to construct calibration curves. Each calibration experiment was performed in triplicate, while recovery measurements were conducted in quintuplicate to ensure statistical reliability. Serum recovery studies were conducted to evaluate the accuracy of the developed sensor, in which known amounts of pure GLB standard solution were spiked into commercial serum samples. DPV was employed for all measurements, and the corresponding GLB concentrations were determined using the previously established regression equation.

## Results and discussion

### Surface characterization of nanomaterials

**Figure 1.**
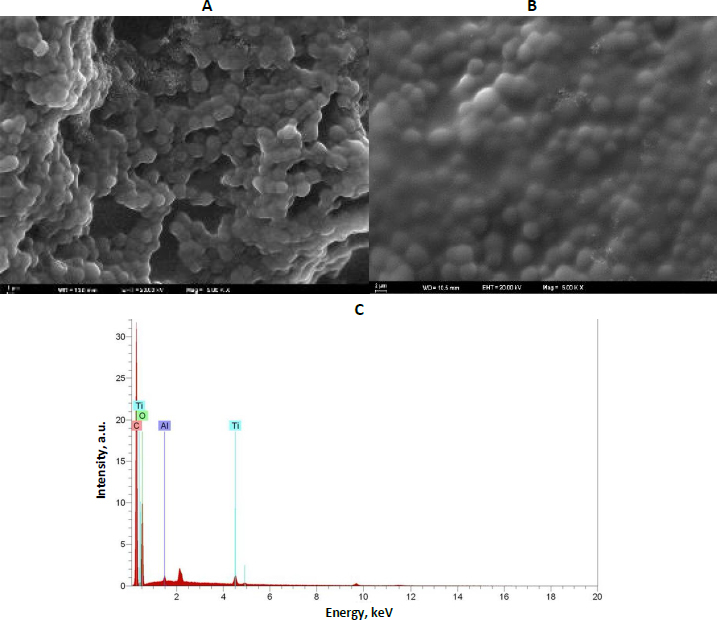
The surface characterization of the electrode. SEM images of (A) GLB/4-ABA@TiO_2_ NPs/MIP-GCE, (B) GLB/4-ABA@TiO_2_ NPs/NIP-GCE, (C) EDX spectra of the GLB/4-ABA@TiO2 NPs/MIP-GCE sensor

The surface morphology of the fabricated sensors was systematically examined using SEM to elucidate the structural differences between MIP and NIP films (Figure 1). SEM images revealed that the GLB/4-ABA@TiO_2_ NPs/MIP-GCE sensor exhibited a notably rough and highly porous surface following the removal of the GLB template (Figure 1A). These three-dimensional cavities correspond to the voids created by GLB extraction, providing a larger effective surface area and facilitating enhanced analyte accessibility. In contrast, the NIP surfaces displayed a relatively smooth, compact morphology, lacking defined cavities, underscoring the critical role of template-directed polymerization in generating selective binding sites (Figure 1B). SEM analyses confirmed that the surface of the GLB/4-ABA@TiO_2_ NPs/MIP-GCE sensor had a rough, porous structure, as predicted for MIPs, whereas the NIP surfaces exhibited a smoother, more uniform structure. These findings support the role of porous MIP structures in enhancing the sensor's selectivity and binding efficiency. EDX spectroscopy was used to investigate the elemental composition further and confirm the successful incorporation of TiO_2_ NPs into the polymeric matrix. The EDX spectra showed characteristic signals for titanium (Ti) and oxygen (O), uniformly distributed across the electrode surface, confirming homogeneous integration of the nanoparticles. The combined SEM and EDX analyses indicate that the MIP-based sensor possesses a highly porous architecture and homogeneously dispersed conductive nanomaterials, providing a favourable environment for selective recognition and sensitive electrochemical detection of GLB. In addition, the EDX spectra of the GLB/4-ABA@TiO_2_ NPs/MIP-GCE sensor confirmed the elemental composition of the polymer matrix, with the presence of Ti, O, Cl, and Al indicating successful incorporation of TiO_2_ NPs and supporting the expected chemical structure of the fabricated sensor (Figure 1C).

### Electrochemical characterization of the GLB/4-ABA@TiO_2_ NPs/MIP-GCE sensor

**Figure 2.**
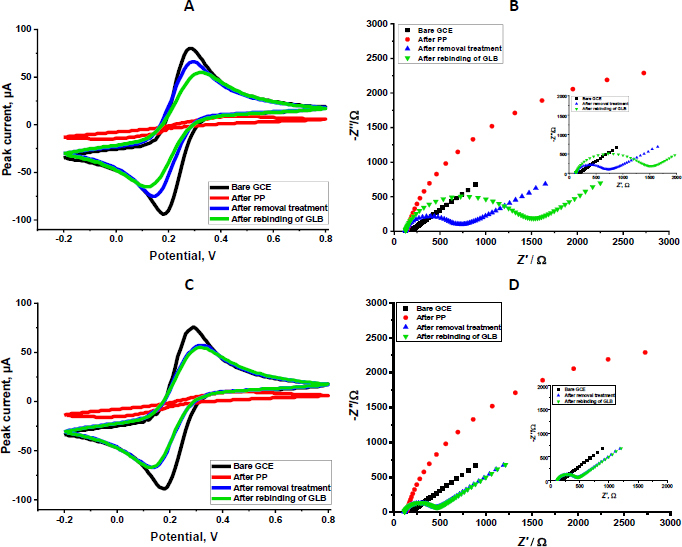
CV of GLB/4-ABA@TiO_2_ NPs/MIP-GCE (A) and GLB/4-ABA@TiO_2_ NPs/NIP-GCE (C); EIS Nyquist plots of GLB/4-ABA@TiO_2_ NPs/MIP-GCE (B) and GLB/4-ABA@TiO_2_ NPs/NIP-GCE (D); (5.0 mM [Fe(CN)_6_]^3−/4−^ solution (CV potential scan range: -0.2 to +0.8 V, scan rate: 0.05 V s^-1^, step potential: 0.01 V; EIS frequency: 0.1 to 100,000 Hz, *E*_ac_ = 0.01 V

The electrochemical behaviour of the developed sensor was investigated by CV and EIS with a 5.0 mM [Fe(CN)_6_]^3−/4−^ redox probe (Figure 2). The analyses were conducted at four key stages of sensor fabrication and operation: the bare GCE surface, post-polymerization, after template removal, and following rebinding of the target molecule GLB. CV measurements showed a well-defined redox couple at the bare GCE, indicating efficient electron transfer. According to the CV results, the bare GCE exhibited the highest peak currents, providing a surface suitable for rapid electron transfer. Following polymerization, a substantial decrease in peak currents was observed, reflecting the formation of the polymeric film that partially hindered electron transfer. After GLB removal, the currents increased due to the formation of imprinted cavities, which facilitated improved diffusion of the redox probe to the electrode surface. Upon rebinding GLB, the peak currents decreased again, confirming the selective occupancy of the imprinted sites by the target molecule (Figure 2A). Complementary EIS analyses corroborated these observations. The bare GCE exhibited a minimal charge-transfer resistance (*R*_ct_ = 62.3 Ω), consistent with rapid electron exchange. After polymerization, *R*_ct_ (104 kΩ) significantly increased due to the insulating nature of the polymer film. Removal of the template resulted in a marked decrease in *R*_ct_ (758 Ω), indicating the formation of conductive pathways within the porous MIP structure. Subsequent rebinding of GLB led to a measurable increase in *R*_ct_ (1320 Ω), further confirming the selective recognition and binding of the analyte.

The NIP-GCE sensor, fabricated under identical conditions but without the GLB template, exhibited markedly different electrochemical characteristics. CV measurements revealed that, while the bare GCE displayed a well-defined redox couple with high peak currents, the NIP sensor showed a decrease in peak currents after polymerization due to the formation of a polymer layer. Unlike the MIP sensor, however, subsequent “template removal” had a negligible impact on the peak currents in the NIP, as no specific recognition cavities were present to enhance the diffusion of the redox probe. Similarly, upon exposure to GLB, the NIP sensor exhibited only minimal current changes, highlighting the absence of selective binding sites (Figure 2C). In contrast, the MIP sensor displayed significant modulation of peak currents at each stage, reflecting the formation and occupancy of selective binding sites. EIS analyses further confirmed this behaviour. The bare GCE exhibited a low charge transfer resistance (*R*_ct_, 44.3 Ω), whereas both MIP- and NIP-polymerized electrodes showed an increase in *R*_ct_ (114 kΩ) due to the insulating polymer matrix. After template removal, *R*_ct_ (508 Ω) decreased substantially in the MIP sensor, indicating the creation of conductive channels within the imprinted cavities. In contrast, the NIP sensor exhibited only a minor decrease, consistent with the absence of specific voids.

Upon GLB rebinding, *R*_ct_ (608 Ω) increased sharply in the MIP sensor, reflecting selective occupancy of the imprinted sites. In contrast, the NIP sensor exhibited only negligible changes, confirming the absence of molecular recognition (Figure 2D).

In addition, the Randles-Ševčik equation (*I*_p_ = 2.69×10^5^*n*^3/2^*AD*^1/2^*ν* ^1/2^*C*) [18] was used to calculate the electroactive surface areas of GCE at all polymerization stages. In this equation, *I*_p_ stands for the peak current, *n* stands for the number of transferred electrons (calculated as 1 for potassium ferri/ferrocyanide), *A* / cm^2^ stands for the active surface area (), *D* stands for the diffusion coefficient (calculated as 7.6×10^−6^ cm^2^ s^−1^ for potassium ferri/ferrocyanide), *ν* stands for the scan rate, and *C* stands for the concentration of probe. According to the results, the electroactive surface areas of GCE before polymerization, after polymerization, after removal, and after rebinding were 0.067, 0.0016, 0.055 and 0.045 cm^2^, respectively.

### Optimization of GLB/4-ABA@TiO_2_ NPs/MIP-GCE sensor production

#### Type and amount of nanomaterials

**Figure 3.**
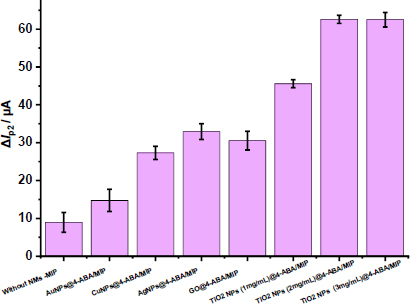
Plot of Δ*I*_p2_ values for the different nanomaterials used to prepare MIP-based sensor obtained by DPV in KCl with 5.0 mM [Fe(CN)_6_]^3−/4−^. (potential scan range, -0.2 to +0.8 V; scan rate, 1.587 mV s^-1^; step potential 8 mV; modulation amplitude 50 mV; modulation time 0.05 s and interval time 0.5 s)

Nanomaterials, owing to their high electrical conductivity and large specific surface area, can significantly enhance electron transfer in electrochemical systems. The influence of various nanomaterials on the performance of the GLB/4-ABA@TiO_2_ NPs/MIP-GCE sensor was systematically evaluated using DPV, by comparing the changes in peak currents (Δ*I*_p2_) before and after template removal in the presence of a 5.0 mM [Fe(CN)_6_]^3−/4−^ redox probe (Figure 3). Five types of nanomaterials were investigated: AuNPs, CuNPs, AgNPs, GO, and TiO_2_ NPs at varying concentrations (1, 2 and 3 mg mL^-1^). The MIP sensor without nanomaterials exhibited a Δ*I*_p2_ of 10 μA, whereas incorporation of TiO_2_ NPs resulted in markedly higher Δ*I*_p2_ values, indicating improved sensor performance. Notably, negligible differences were observed between the 2 and 3 mg mL^-1^ TiO_2_NPs-modified sensors, suggesting that 2 mg mL^-1^ was sufficient to achieve optimal enhancement. Although MIP sensors modified with the other nanomaterials (AuNPs, CuNPs, AgNPs, and GO) displayed higher Δ*I*_p2_ values relative to the unmodified sensor, their Δ*I*_p2_ responses were comparatively lower, demonstrating less effective template site accessibility and recognition. Based on these observations, TiO_2_NPs at 2 mg mL^-1^ were selected for further sensor fabrication and subsequent experiments, as they provided the most favourable balance between conductivity enhancement and template recognition efficiency (Figure 3).

#### Monomer/template ratio

**Figure 4.**
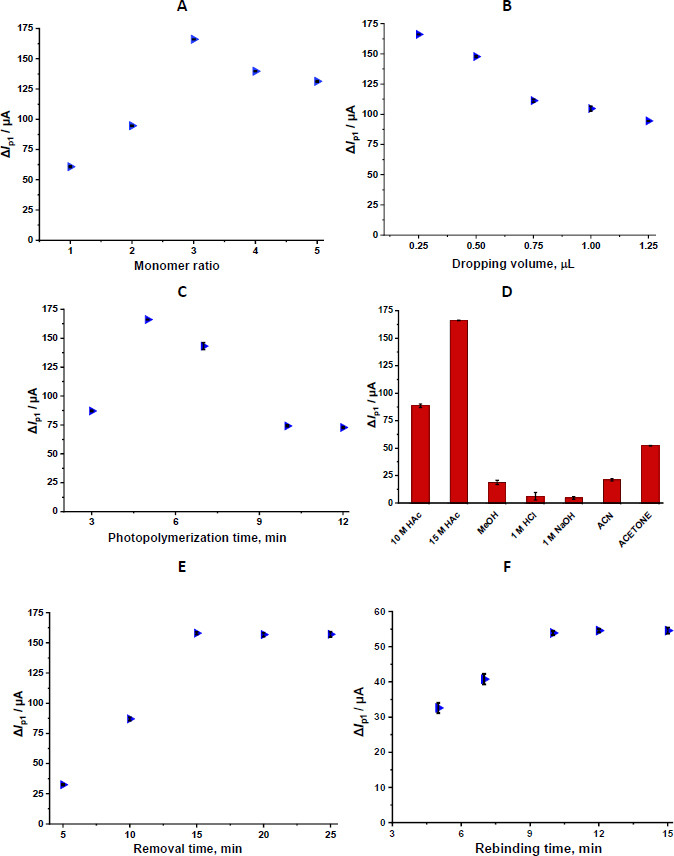
Optimization of parameters for GLB/4-ABA@TiO_2_ NPs/MIP-GCE sensor. (A) monomer to template ratio. (B) the dropping volume of the solution. (C) PP time. (D) removal solutions. (E) removal time. (F) rebinding time (Conditions for DPV: potential scan range, -0.2 to +0.8 V; scan rate, 1.587 Vs^-1^; step potential 8 mV; modulation amplitude 50 mV; modulation time 0.05 s; and interval time 0.5 s)

The appropriate ratio between the template molecule and the functional monomer is a critical parameter for increasing selectivity in MIP synthesis. A low concentration of functional monomer can prevent the formation of sufficient recognition cavities. In contrast, excessive monomer use can lead to the formation of random binding sites rather than the orderly formation of target-specific cavities. Therefore, different monomer: template molar ratios were systematically evaluated. Ratios between 1:1 and 1:5 were tested, and the peak current differences (Δ*I*_p1_) obtained after polymerization and template removal under each condition were analysed. The findings show that the highest Δ*I*_p1_ value was obtained at a 1:3 ratio, indicating optimal sensor performance (Figure 4A).

#### Dropping volume

The morphology and electrochemical performance of the MIP layer are directly dependent on the volume of the drop used during polymerization. Dropping volume is considered a determining factor in the thickness, conductivity, and polymerization efficiency of the polymer layer. To ensure a uniform coating on the GCE surface and prevent the formation of an excessively thick layer, different volumes (0.25 to 1.25 μL) of prepolymer solutions were applied. The differences in peak current (Δ*I*_p1_) obtained after template removal and polymerization were evaluated for each drop volume. The results showed that the highest Δ*I*_p1_ was achieved at a volume of 0.25 μL, indicating optimal sensor coverage. The decrease in ΔIp_1_ at higher volumes is attributed to increased layer thickness, which hinders ion transfer (Figure 4B). Accordingly, this dropping volume was selected for subsequent sensor fabrication to maximize sensitivity and reproducibility.

#### Photopolymerization time

PP time is a critical parameter that determines the thickness and structural properties of the MIP layer formed on the electrode surface. Because the duration of surface exposure to UV light directly affects polymerization efficiency, this time should be carefully optimized. In the study, 0.25 μL of prepolymer solution was applied, and the electrode surface was exposed to UV light (365 nm, 100 W) for 3, 5, 7, 10 and 12 min. Polymerization efficiency was evaluated by considering the Δ*I*_p1_ values. The findings revealed that a 5-minute PP time provided the highest and most reproducible ΔIp_1_. As shown in Figure 4C, prolonged polymerization time results in the formation of a denser and more compact polymer layer, which enhances the film’s mechanical stability but simultaneously hinders template removal and diminishes the accessibility and efficiency of the selective recognition cavities (Figure 4C).

#### Removal solution and removal time

The removal of the template molecule under optimal conditions represents a critical step in the fabrication of MIP-based sensors, as it directly determines the formation and accessibility of selective recognition sites. During this stage, the target molecule (GLB) is extracted from the polymer matrix, thereby generating analyte-specific cavities that are complementary in shape, size, and functional group orientation. These cavities are essential for ensuring structural compatibility and high molecular selectivity during subsequent rebinding processes. To identify the most efficient removal medium without compromising the structural integrity of the polymer network, various solvents and chemical agents were systematically evaluated. The solvents tested were 1 M NaOH, 1 M HCl, MeOH, ACN, acetone, and HAc (10 M and 15 M). The efficiency of each extraction medium was assessed by comparing the Δ*I*_p1_ values. Among the tested solvents, 15 M HAc produced the most outstanding Δ*I*_p1_ value, indicating the most effective removal of the GLB template from the polymeric matrix. This suggests that concentrated acetic acid provides the optimal balance between strong solvation capability and preservation of the polymeric architecture, thereby maximizing the accessibility of imprinted cavities for subsequent rebinding of the target analyte (Figure 4D).

After selecting the most effective extraction solution, the template removal time was optimized to remove the GLB template while preserving the structural integrity of the imprinted polymer. Removal times ranging from 5 to 25 min were systematically investigated under identical experimental conditions, and the corresponding Δ*I*_p_ values were used to assess extraction efficiency. The results demonstrated that shorter removal times (5-10 min) were insufficient for complete template removal, as evidenced by lower Δ*I*_p_ values, likely due to the incomplete desorption of GLB molecules from the imprinted cavities. Among the tested durations, a 15-minute extraction time produced the highest and most reproducible current difference, indicating efficient removal of the template molecule without compromising the physical or electrochemical stability of the MIP film. Consequently, a 15-minute extraction period was established as the optimal condition and was employed in all subsequent experiments (Figure 4E).

#### Rebinding time

One of the critical parameters determining MIP sensor performance is rebinding time. In this study, different time periods (5, 7, 10, 12, and 15 min) were tested to evaluate the sensor's ability to rebind to the target analyte after template removal. During the experiments, the sensor was immersed in an analyte solution of the specified concentration and operated at 250 rpm on a Thermo-shaker at 25 °C. Rebinding efficiency was calculated from the peak current difference (Δ*I*_p2_) measured after removal and binding. The findings showed that no significant change was observed in Δ*I*_p2_ values after 10 min, while the results remained stable over extended periods. Therefore, a rebinding time of 10 min was selected as the optimum condition (Figure 4F).

### Analytical performance of the GLB/4-ABA@TiO_2_ NPs/MIP-GCE sensor in standard solutions

**Figure 5.**
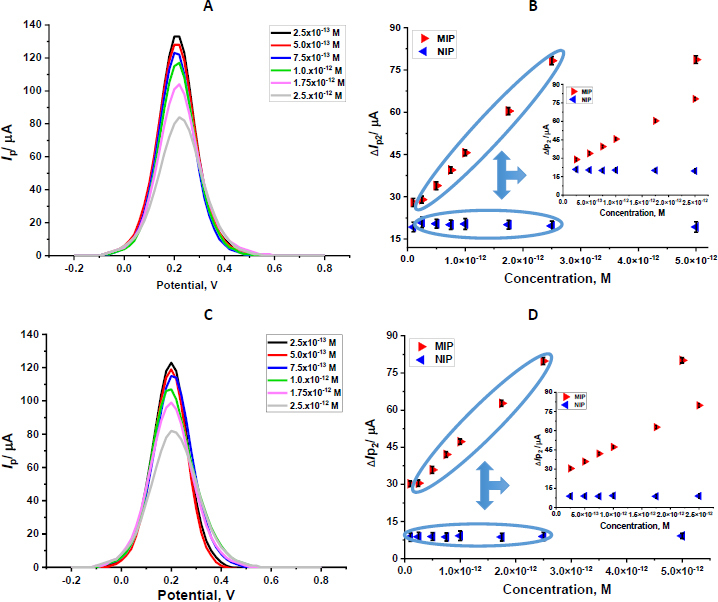
DP voltammograms obtained after rebinding of various GLB concentrations in standard solutions (A) and commercial serum solutions (C). Calibration curve for GLB/4-ABA@TiO_2_ NPs/MIP-GCE in standard solutions (B) and commercial serum solutions (D) (in 5.0 mM [Fe(CN)_6_]^3−/4−^ solution in the presence of 0.1 M KCl); concentration range 2.5×10^-13^ to 2.5×10^-12^ M of GLB. The measurements were performed in 5.0 mM [Fe(CN)_6_]^3−/4−^ solution (potential scan range -0.2 to +0.8 V; scan rate 1.587 mV s^-1^; step potential 8 mV; modulation amplitude 50 mV; modulation time 0.05 s; interval time, 0.5 s)

To evaluate the analytical performance of the fabricated GLB/4-ABA@TiO_2_ NPs/MIP-GCE sensor, a systematic study was conducted to determine its linear dynamic range for GLB detection under optimized experimental conditions. DPV was employed as an analytical technique, using a 5.0 mM [Fe(CN)_6_]^3-/4-^ redox probe to indirectly monitor the sensor response. As illustrated in Figure 5A, the anodic peak current of the redox probe decreased gradually with increasing GLB concentration, attributed to the progressive occupation of the imprinted recognition sites by GLB molecules, hindering electron transfer at the electrode surface. Quantitative analysis revealed a well-defined linear correlation between the measured current differences (Δ*I*_p2_) and GLB concentrations over the range of 2.5×10^-13^ to 2.5×10^-12^ M (Figure 5B). The corresponding regression equation was found to be Δ*I*_p2_ = 2.17×10^13^*C* + 23.37, with an excellent correlation coefficient (*R*^2^ = 0.998). The limit of detection (LOD) and limit of quantification (LOQ) were calculated following the International Council for Harmonisation (ICH) guidelines using the equations LOD = 3σ / *m* and LOQ = 10σ / *m*, where σ is the standard deviation and *m* is the slope [19,20].

Here, the standard deviation was calculated from the average of three replicate measurements at the lowest concentration on the calibration curve. The resulting regression and validation parameters are summarized in Table 1. To verify the specificity of the molecular imprinting process, a NIP sensor was fabricated under identical conditions but in the absence of the GLB template. The NIP electrode exhibited negligible changes in peak current upon successive additions of GLB, and no linear relationship was observed between ΔIp_2_ and GLB concentration (Figure 5B).

**Table 1.** Regression data of the calibration line for GLB/4-ABA@TiO_2_ NPs/MIP

Parameter	Standard solution	Serum sample
Linearity range, M	2.50×10^-13^ to 2.50×10^-12^	2.50×10^-13^ to 2.50×10^-12^
Slope, μA M^−1^	2.17×10^13^	2.13×10^13^
Standard error of slope	3.74×10^11^	2.57×10^11^
Intercept, μA	23.37	26.20
Standard error of intercept	0.48	0.66
Correlation coefficient (*r*)	0.998	0.999
LOD, M	3.62×10^-14^	6.44×10^-14^
LOQ, M	1.21×10^-13^	2.15×10^-13^
Repeatability of peak current RSD, %*	0.89	0.97
Reproducibility of peak current RSD, %*	1.42	1.76

*Each value is the mean of three experiments

Overall, the GLB/4-ABA@TiO_2_ NPs/MIP-GCE sensor demonstrated outstanding analytical performance, characterized by high sensitivity, excellent selectivity, and remarkable reproducibility. These results highlight its potential as a powerful analytical platform for trace-level detection of GLB in clinical and pharmaceutical applications.

To confirm the precision of the created MIP sensor, a NIP sensor was fabricated without GLB under identical conditions, and no notable variations in peak current were observed in DPV recordings with varying GLB concentrations. In contrast to the MIP sensor, the NIP sensor failed to show a linear response (Figure 5B), demonstrating the MIP sensor's specificity for GLB. Additionally, this highlights the crucial role of the GLB template in enhancing the sensor's performance.

Moreover, electrochemical MIP sensors employ an indirect approach for highly sensitive detection of target analytes at low concentrations (pM and fM). Quantification of the target analyte is based on the difference (Δ*I*_2_) between the peak current values obtained after removal and rebinding. This approach is based on monitoring changes in the electrochemical response of a redox probe ([Fe(CN)_6_]^3-/4-^) as it diffuses to the electrode surface. Upon rebinding of the analyte at specific recognition sites within the MIP layer, ion transport and electron transfer at the electrode interface are restricted, resulting in a measurable decrease in current or an increase in charge-transfer resistance. The resulting signal is inversely proportional to analyte concentration, making the indirect method highly selective and sensitive for detecting target analytes.

### Applicability in biological samples and pharmaceutical formulations

Biological samples and pharmaceutical formulations were used to evaluate the applicability of the developed sensor. Under these conditions, the GLB/4-ABA@TiO_2_ NPs/MIP-GCE sensor exhibited a linear response over the concentration range of 2.50×10^−13^ to 2.50×10^−12^ M of GLB, which could be adjusted to the following regression equation: Δ*I*_p2_ = 2.13×10^13^*C* + 26.20 (*R*^2^ = 0.999)

The LOD and LOQ values in serum samples were calculated to be 6.44×10^-14^ and 2.15×10^-13^ M, respectively (Table 1). Figure 5C presents the DP voltammograms obtained in serum, while Figure 5D illustrates the corresponding calibration curves. To assess the sensor’s accuracy, serum samples were spiked with GLB standard solutions at known concentrations. The calculated recovery percentages and their respective relative standard deviations (RSD, %) were within acceptable limits, confirming the method’s reliability (Table 2). Moreover, the calibration slopes obtained in the serum matrix were comparable to those derived from standard solutions, indicating minimal matrix interference. Pharmaceutical dosage form analyses were performed to validate the sensor’s practical applicability. GLB was incorporated into capsule formulations, and recovery experiments were conducted to evaluate potential matrix effects from common excipients. The high recovery rates and low RSD values (Table 3) demonstrate the method’s accuracy, precision, and suitability for routine pharmaceutical quality control.

**Table 2.** Recovery experiment results for commercial human serum samples

Parameter	Commercial serum sample		
Sample concentration, M	0.50×10^-12^	0.75×10^-13^	1.00x10^-12^
Spiked amount, M	1.25×10^-12^	1.00x10^-12^	0.75×10^-12^
Found amount, M*	1.72×10^-12^	1.75×10^-12^	1.73×10^-12^
Average recovery, %*	99.18	99.81	99.64
RSD of recovery, %	1.52	1.29	1.58
Bias, %	+0.72	+0.19	+0.36

*Each value is the mean of five experiments

**Table 3.** Recovery experiment results for tablet samples

Parameter	Tablet dosage form
Label amount, μg	630.00
Found amount, μg*	631.30
RSD, %	2.63
Spiked amount, mg	10.00
Found amount, mg*	10.05
Average recovery, %*	100.51

*Each value is the mean of five experiments

### Selectivity studies

To evaluate the selectivity of the developed sensor toward GLB in the presence of structurally related compounds, the imprinting factor (*k*) and relative imprinting factor (*k*′) were determined. The selective recognition ability of the MIP arises from the formation of specific binding cavities within its structure, which are spatially and chemically complementary to the template molecule, GLB. To investigate potential cross-reactivity, several structurally analogous compounds, otilonium bromide (OTI), aclidinium bromide (ACL), tiotropium bromide (TIO), oxitropium bromide (OXI), and ipratropium bromide (IPR), were selected as test analytes. The calculated imprinting and relative imprinting factors, summarized in Table 4, quantitatively describe the selective binding performance of the MIP sensor. The results revealed that the GLB/4-ABA@TiO_2_ NPs/MIP-GCE sensor exhibited a significantly higher affinity for GLB compared to the structurally analogous compounds, with selectivity factors of 4.34, 4.30, 4.29, 4.30 and 4.17 for OTI, ACL, TIO, OXI, and IPR, respectively. These findings confirm the high molecular recognition capability and specificity of the MIP-based sensor, primarily due to the complementary size, shape, and functional-group orientation of the imprinted cavities formed during polymerization.

**Table 4.** Specificity of GLB/4-ABA@TiO_2_ NPs/MIP-GCE for the determination of GLB

Molecules	MIP/GCE		NIP/GCE		*k*'_(MIP/NIP)_
	Δ*I*_2_ / μA	*k*_(MIP)_	Δ*I*_2_ / μA	*k*_(NIP)_	
GLB	44.62	-	9.55	-	-
OTI	16.34	2.73	15.2	0.6	4.34
ACL	13.65	3.26	12.58	0.75	4.30
TIO	13.66	3.26	12.55	0.76	4.29
OXI	13.71	3.25	12.62	0.75	4.30
IPR	12.69	3.51	11.33	0.84	4.17

**Figure 6.**
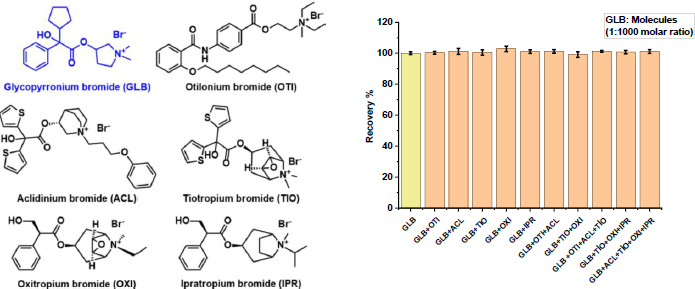
The response of GLB/4-ABA@TiO_2_ NPs/MIP-GCE sensor to 10^-12^ M of GLB in the presence of 10^-8^ M of OTI, ACL, TIO, OXI and IPR

This research also included the examination of Δ*I*_p2_ current values using DPV for GLB in the presence of OTI, ACL, TIO, OXI, and IPR. The findings revealed that the created sensor displayed remarkable selectivity, even when subjected to concentrations of structurally analogous compounds that were 1000 times greater (Figure 6).

### Interference study

**Figure 7.**
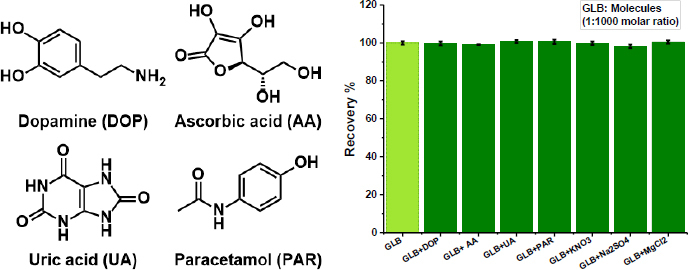
Recovery studies in the detection of GLB in the presence of other compounds

To demonstrate the reliability of the developed sensor in biological environments, several compounds that could affect measurement results were examined. For this purpose, KNO_3_, MgCl_2_, Na_2_SO_4_, dopamine (DOP), ascorbic acid (AA), uric acid (UA), and paracetamol (PAR), which are commonly found in body fluids, were selected. The concentrations of these substances in the prepared solutions increased to 1000 times the concentration of the target analyte, and the sensor response was evaluated. DPV measurements yielded recovery percentages ranging from 98.18 to 100.75 %, with RSDs of less than 1.73 %. The findings demonstrated that these compounds, even at very high concentrations, did not significantly affect the sensor's accuracy and selectivity. Thus, it was confirmed that the GLB/4-ABA@TiO_2_ NPs/MIP-GCE sensor can be used reliably for determining GLB in biological samples (Figure 7).

### Stability tests

To examine the stability of the GLB/4-ABA@TiO_2_ NPs/MIP-GCE sensor, the electrode was stored in a desiccator at room temperature for 7 days. The evaluations revealed that the sensor's performance was 90.29 % by the end of the third day, 82.52 % by the end of the fifth day, and 70.87 % by the end of the seventh day. These findings indicate that the developed sensor can be used stably for approximately 5 days.

### Comparison with other methods

A summary of the analytical techniques previously used to evaluate GLB performance is presented in Table 5. However, many of these approaches rely on expensive or hazardous reagents, require extensive sample-pretreatment procedures, and involve long analysis times, making them less practical for routine use. Moreover, the linear range, detection limit, and real-world sample applications of the fabricated sensor are comparable to those found in the literature. The GLB/4-ABA@TiO_2_ NPs/MIP-GCE platform demonstrated highly sensitive and selective determination of GLB in real matrices when compared with conventional methods. Overall, the results confirm that this strategy offers a simple, eco-friendly, cost-effective, and practical alternative by minimizing solvent consumption and operational complexity. The findings further highlight the sensor’s excellent linearity, repeatability, reproducibility, low detection limit, selectivity, and stability relative to existing analytical techniques.

**Table 5.** Comparison of previous studies on GLB determination with this study

Method	Linear range	LOD	Sample	Recovery, %	Ref.
HPLC	-	3.5 ng mL^-1^	Drug	-	[5]
RP-HPLC/UV	20 to 120 μg mL-1	4.00 μg mL^-1^	Drug	99.11	[8]
UHPLC-HESI-MS-MS	0.125 to25 pg mL^-1^	0.025 pg mL^-1^	Horse plasma	78.00 to 96.00	[21]
IPC	0.3 to 30.0 μg mL^-1^	0.074 μg mL^-1^	Inhaler capsule	99.87	[22]
CE	20 to 800 ng mL^-1^	2.84 ng mL^-1^	Inhaler capsule	93.56	[23]
GLB/4-ABA@TiO_2_ NPs/MIP-GCE	2.5×10^-13^ to 2.5×10^-12^ M	3.62×10^−14^	Capsule	100.51	This study

HPLC: High-performance liquid chromatography, RP-HPLC/UV: UHPLC-HESI-MS-MS: Ultra-high-performance liquid chromatography with heated electrospray ionization-tandem mass spectrometry, IPC: Ion-pairing chromatography, CE: Capillary electrophoresis

## Conclusion

This study is the first to investigate the selective and sensitive determination of GLB in various media using a MIP-based electrochemical sensor. The GLB/4-ABA@TiO_2_ NPs/MIP-GCE sensor has been successfully applied to both biological samples and pharmaceutical formulations, thanks to its short analysis time, high sensitivity and selectivity, and practical use. The sensor demonstrated excellent linearity for GLB determination in the range of 2.5×10^-13^ to 2.5×10^-12^ M in standard solutions and biological matrices, achieving low LOD and LOQ values. Recovery studies in commercial serum samples and in pharmaceutical dosage forms of GLB validated the sensor's accuracy. Selectivity evaluations were conducted with structurally similar drugs (OTI, ACL, TIO, OXI, IPR), and it was found that sensor performance was not affected even when the concentrations of these compounds were 1000-fold higher than that of the target molecule. The results demonstrate that the developed sensor is a reliable platform for use in clinical and industrial settings. Its high sensitivity could contribute to drug delivery studies. It is also anticipated that it could be adapted for use in portable diagnostic systems for biomedical research, enabling the detection of very low concentrations of GLB.
